# Use of Poly(vinyl alcohol)-Malic Acid (CLHPMA) Hydrogels and Chitosan Coated Calcium Alginate (CCCA) Microparticles as Potential Sorbent Phases for the Extraction and Quantitative Determination of Pesticides from Aqueous Solutions

**DOI:** 10.3390/polym13223993

**Published:** 2021-11-19

**Authors:** Cristian Valdés, Oscar Valdés, Daniel Bustos, Diana Abril, Gustavo Cabrera-Barjas, Alfredo Pereira, Jorge Villaseñor, Efraín Polo-Cuadrado, Gustavo Carreño, Esteban F. Durán-Lara, Adolfo Marican

**Affiliations:** 1 Centro de Investigación de Estudios Avanzados del Maule (CIEAM), Vicerrectoria de Investigación y Postgrado, Universidad Católica del Maule, Talca 3460000, Chile; cvaldesv@ucm.cl (C.V.); ovaldes@ucm.cl (O.V.); dbustos@ucm.cl (D.B.); 2 Laboratorio de Bioinformática y Química Computacional (LBQC), Facultad de Medicina, Universidad Católica del Maule, Talca 3460000, Chile; 3 Escuela de Bioingeniería Médica, Facultad de Medicina, Universidad Católica del Maule, Talca 3460000, Chile; 4 Departamento de Biología y Química, Facultad de Ciencias Básicas, Universidad Católica del Maule, Talca 3460000, Chile; dabril@ucm.cl; 5 Unidad de Desarrollo Tecnológico (UDT), Universidad de Concepción, Parque Industrial Coronel, Coronel 3349001, Chile; g.cabrera@udt.cl; 6 Instituto de Química de Recursos Naturales, Universidad de Talca, Talca 3460000, Maule, Chile; alpereira@utalca.cl (A.P.); jvillase@utalca.cl (J.V.); epolo@utalca.cl (E.P.-C.); gcarreno@utalca.cl (G.C.); 7 Bio and NanoMaterials Lab, Drug Delivery and Controlled Release, Universidad de Talca, Talca 3460000, Maule, Chile; 8 Departamento de Microbiología, Facultad de Ciencias de la Salud, Universidad de Talca, Talca 3460000, Maule, Chile

**Keywords:** sorbent phase, CLPHMA, hydrogel, chitosan, alginate, molecular dynamics simulation

## Abstract

Pesticides are used worldwide to increase crop yields in agriculture. However, their toxicity and accumulation capacity can make them toxic to the environment, animals and humans. In the case of workers chronically exposed to these substances, they must be sampled continuously, so urine is an excellent option. In this sense, this study proposes to use poly(vinyl alcohol)-malic acid hydrogels, and chitosan-coated calcium alginate as new sorbent phases to be used in pesticide determination processes in urine. To better understand the behavior of these materials in the capture and desorption process, molecular dynamics simulations (MDS) were used, and desorption experiments were performed, using mechanical agitation, ultrasound, and pH variation in the desorption process, in order to optimize the parameters to obtain better recoveries. Under the optimal experimental conditions, the maximum recoveries were of the order of 11% (CFN), 3% (KCF), 53% (DMT), 18% (MTD) and 35% (MTL). Although the recoveries were not exhaustive, they are a first approximation for the use of these new sorbent phases in the determination of this type of compound in aqueous solutions and urine.

## 1. Introduction

The use of pesticides in agriculture helps increase crop yields to meet the demands of the growing human population. However, continuous application can cause chronic abnormalities in humans and destroy the environment and biodiversity [1,2,3,4]. Depending on the chemical structure some pesticides are persistent in the environment and can be accumulated in cattle meat, vegetables and fruits, and eaten by humans [5,6,7,8].

Due to the high toxicity of some pesticides, in terms of their effects on human health due to acute exposure, the sampling in people who show some symptom of poisoning is crucial for an opportune diagnosis. On the other hand, people exposed to these pesticides chronically, due to their work, should be able to be controlled systematically and minimally invasive. In this sense, urine is a good selection as a sample for analysis because it can be obtained in large volumes, being a non-invasive sampling, so that the repetition of sampling is not problematic [9].

Considering the difficulties in sampling and sample treatment, the aim of this work is to evaluate the efficiency of new insoluble materials based on hydrogels, as a potential sorption phase to extract and preconcentrate pesticides and their metabolites from aqueous matrices, and their subsequent desorption for their determination by chromatography. Hydrogels are three-dimensional polymeric networks formed by the cross-linking of monomers or polymeric chains via covalent bonds and/or non-covalent interactions, such as hydrogen bridges, host–guest complexation, electrostatic interactions or their combinations, whose interactions are usually stable [10]. They can be prepared as “smart” materials that are highly responsive to changes in their physical or chemical environment. Hydrogels have the ability to incorporate a large amount of water or biological fluids [11,12].

Among the polymers most used for the preparation of hydrogels are alginate (ALG), chitosan (CT) [13] and poly(vinyl alcohol) (PVA) [14]. Calcium ALG (CaALG) is a salt of alginic acid (anionic polysaccharide) formed by linear copolymer with homopolymeric blocks of (1–4)-linked β-d-mannuronate (M) and its C-5 epimer α-l-guluronate (G) residues, respectively. CT is obtained by the deacetylation of chitin and is a linear cationic polysaccharide composed of β-(1–4)-2-acetamido-2-deoxy-β-d-glucopyranose and 2-amino-2-deoxy-β-d-glucopyranose [15]. PVA is a hydrophilic, biodegradable and biocompatible synthetic polymer that has been widely used in different areas of the biomedical field [16].

ALG and CT have been used in pesticide removal from aqueous solutions [17,18,19,20]. PVA hydrogels have high elasticity and mechanical strength and low cost, and have attracted great attention in wastewater treatment and pesticide capture [21,22,23].

In this study, two groups of insoluble materials based on hydrogels were synthesized and tested for the extraction and preconcentration tests:

(1) Polyelectrolyte complex (PEC), composed of CT and CaALG. The polyelectrolyte complexes result from the interaction of macromolecules that have complementary ionizable electrostatic groups. The use of natural polyelectrolytes to prepare polyelectrolyte complexes is due to their advantages in terms of their low cost and compatibility, and their minimal consumption of organic solvents. They are also safe and stable and have been approved for use in humans [24]. In this way, chitosan-calcium alginate complexes (CCAC), and chitosan-coated calcium alginate microparticles (CCCA) were synthesized.
(2) Hydrogels based on poly(vinyl alcohol) (PVA) crosslinked with malic acid (MA) in different proportions of PVA-MA. These materials have been successfully proven in our study group as superadsorbents for dimethoate and methamidophos for remediation of aqueous solutions [21,23].

The selected compounds were carbofuran (CFN), carbofuran-3-keto (KCF), dimethoate (DMT), methamidophos (MTD) and methomyl (MTL). These compounds were selected considering the wide use and toxicity of these pesticides in agriculture. In addition, a metabolite (KCF) was selected to evaluate its monitoring as a body degradation product in urine.

## 2. Theoretical Section

The five compounds CFN, KCF, DMT, MTD and MTL, and the ALG monomers *α-l-gulopyranuronate* (G), *β-d-mannopyranuronate* (M), CT monomer, the PVA monomer and the MA crosslinker were sketched in Maestro/Schrödinger suite [25]. Their tautomeric forms and isomers were assigned with Ligprep software (Schrödinger Release 2021-4. Schrödinger Inc., New York, NY, USA). Similarly, their pKa values were predicted with Epik tool [26]. In consequence, the protonation state in every molecule was assigned according to its computationally predicted pKa value in three experimental pH values (3, 7 and 9).

### 2.1. ALG-CT Chains

We built two ALG chains of 16 subunits: one with eight G monomers followed by eight M monomers named G8-block/M8-block. The second chain involved eight G monomers interspersed by eight M monomers termed G8M8-block. Besides this, one chain of 16 monomers of CT molecule was built (CT8-block). All the chains were assembled in Maestro/Schrödinger suite [25].

### 2.2. PVA-MA Substructures

Considering the experimental proportions of monomer and crosslinker, the LEAP program from AmberTools21 suite [27] was used to build substructures/sheets of PVA-MA hydrogels. Every PVA-MA substructure was constituted by five chains, each of 25 PVA monomers; however, the quantity and distribution of the crosslinkers in each substructure were modified according to Figure S1 in the Supplementary Material. Briefly, 4, 8 and 12 MA were used for the 10, 20 and 30% crosslinker ratios, and in addition two MA distribution patterns were used for each crosslinker ratios. Steric hindrance issues of the substructures were fixed by a short minimization using ‘obminimize’ from Open Babel software version 3.0.0 (University College Cork Co., Cork, Ireland) [28].

### 2.3. ALG-CT and PVA-MA Particles

ALG, CT, and PVA-MA structures were subjected to an energy minimization in an implicit solvent with OPLS v.2005 as force field [29] by a maximum of 5000 iterations. Based on their pKa values, three main configurations of ALG-CT were explored: at pH = 3.0, each ALG monomer had a partial charge of 0, and each CT monomer had a partial charge of +1. At pH = 7, each ALG monomer had a partial charge of −1, and each CT monomer had a partial charge o < f + 1. Finally, at pH = 9, each ALG monomer had a partial charge of −1, and each CT monomer had a partial charge of 0. Subsequently, ALG and CT chains were used as starting point to generate three particles (one per pH value) of ALG-CT, with 20 chains of G8-block/M8-block, 20 chains of G8M8-block and 160 ions of calcium, randomly distributed in a sphere of 55 Å, and 40 chains of CT randomly distributed in a sphere of 60 Å. Ten molecules of each pesticide were randomly placed within the ALG-CT particle in a radius <40 Å. Each molecule was positioned with PACKMOL software v.20.3.1 (University of Campinas, Campinas, Brasil) [30] with a tolerance radius of 4.0 Å between atoms not belonging to the same molecule.

Because our pKa predictions did not detect changes in PVA monomers, here, three configurations were explored based on three crosslinker proportions. Ten sheets of each crosslinker configuration were randomly positioned within a sphere of radius 55 Å. Similar to ALG-CT particles, ten pesticides were incorporated into each particle in a radius <40 Å with PACKMOL software v.20.3.1 [30].

### 2.4. Molecular Dynamics Simulations (MDS)

The three particles of ALG-CT and three particles of PVA-MA were minimized as had been previously undertaken for monomers. Subsequently, each particle was embedded into a periodic boundary box of methanol, and Na^+^ and Cl^−^ counterions were added to neutralize each system if applicable. Each system reached almost 390,000 atoms, which were evaluated through 100 ns of MDs in an NPT ensemble (*p* = 1 atm, T = 293 K) using Desmond and Maestro/Schrödinger suite with OPLS v.2005 as force field [29]. Once the simulations were finished, we evaluated the liberation of each pesticide from the particle, using as a criterion of liberation a distance greater than 5 Å between the pesticide molecule and the particle. Additionally, we computed the diffusion coefficient of each pesticide from the ensemble-averaged squared displacement during the entire simulation time [31]. The radius of gyration was calculated for ALG-CT and PVA-MA particles to evaluate the maintenance or segregation of the chains/sheets that form the particle through the trajectory.

## 3. Experimental

### 3.1. Reagents

Carbofuran (CFN, 99.53%), carbofuran-3-keto (KCF, 94.84%), dimethoate (DMT, 99.37%), methamidophos (MTD, 99.72%) and methomyl (MTL, 99.9%) were purchased at Dr. Ehrenstorfer GmbH (Wesel, Germany). HCl and methanol (HPLC grade) were purchased at PanReac AppliChem (Darmstadt, Germany). Poly(vinyl alcohol) (PVA) 30–60 KDa, malic acid (MA), sodium bicarbonate (NaHCO_3_, ≥99.5%), sodium alginate (NaALG), calcium chloride (CaCl_2_, anhydrous ≥ 97%) and chitosan (CT) were purchased from Sigma-Aldrich (St. Louis, MO, USA). The aqueous solutions were made in MilliQ water.

### 3.2. Synthesis and Characterization of Materials

#### 3.2.1. Synthesis of Hydrogels Based on Poly(vinyl alcohol) (PVA) Crosslinked with Malic Acid (MA) (CLPHMA)

Three CLPHMA hydrogels with different degrees of crosslinking were synthesized. The synthesis was carried out by esterification of PVA with MA, according to the methodology of Schanuel et al. [32]. The reactions were performed by mixing 10 wt.% aqueous solution of PVA with an aqueous solution of MA, in the presence of 1 × 10^−1^ mol L^−1^ HCl (pH 1). The final MA concentrations were 10, 20 and 30 wt.% for CLPHMA-10, CLPHMA-20 and CLPHMA-30 hydrogels, respectively. The reactions were carried out under reflux at 100 °C in a necked flask, with magnetic stirring. After 3 h, the contents of the reaction flask were placed in an oven at 70 °C for 3 h to complete the crosslinking reaction. The CLPH-MA hydrogels were washed three times with NaHCO_3_ to remove excess acid. Finally, the hydrogels were lyophilized to obtain cryogels [33].

#### 3.2.2. Synthesis of Microparticles Based on Polyelectrolyte Complexes (PEC), Composed of CT and CaALG: Calcium Alginate-Chitosan Complexes (CCAC), and Chitosan-Coated Calcium Alginate Microparticles (CCCA)

Microparticles of chitosan-coated calcium alginate (CCCA) were prepared using chitosan/sodium alginate as a coat material employing an ionotropic/external gelation technique similar to that described by Gonzalez-Rodriguez et al. [34], with slight modifications. Ionotropic gelation can be applied to prepare microparticles using combinations of CT and Ca^2+^ as cationic components and ALG as an anion. First, NaALG will be dissolved in distilled water (pH 6.5) at a concentration of 1.0 wt.%. After mixing well, the NaALG solution is dropped using a hypodermic syringe drop-wise at 60 drops/min into 500 mL of 0.1 M calcium chloride aqueous solution under magnetic stirring to form microparticles of calcium alginate (CaALG). Subsequently, the CaALG microparticles thus formed are washed with distilled water and transferred to a CT solution at a concentration of 1% (*w*/*v*) in 1% (*w*/*v*) aqueous acetic acid under gentle magnetic stirring for 10 min at room temperature. Finally, the chitosan–calcium alginate complex (CCAC) microparticles are recovered by filtration, washed with distilled water and dried at room temperature. The optimum conditions for production of the CaALG microparticles were used to prepare CCCA microparticles. In the case of CCCA microparticles, CaCl_2_ was dissolved in 50 mL 1% (*w*/*v*) acetic acid aqueous solution containing 1% (*w*/*v*) CT. Finally, by varying the Ca^2+^ concentration, three formulations were obtained, with different degrees of crosslinking, corresponding to 47.3%, 52.4% and 56.5% for CCCA-47.3, CCCA-52.4 and CCCA-56.5, respectively.

#### 3.2.3. Swelling Studies

CLPHMAs (0.4 to 0.5 mm thick discs, 1 cm in diameter) and CCCAs (about 6 mg) were allowed to swell in distilled water (pH 5.5) at 20 °C. The swollen gels were removed from the water at regular time intervals and dried superficially with filter paper, weighed and placed in the same distilled water. The measurements were continued until constant weight was reached (Equation (1))

(1) $$\%W=\frac{M_{h}-M_{x}}{M_{h}}\times 100$$

where *%W* is the swelling index, *M_h_* and *M_x_* are the masses of the swollen hydrogel and cryogel, respectively.

#### 3.2.4. Characterization of Synthesized Materials by Infrared Spectroscopy and Thermo-Gravimetric Analysis

CLPHMA hydrogels were characterized by Fourier-transform infrared spectroscopy (FT-IR) (Nicolet Nexus 470 FT-IR, Thermo Scientific, Waltham, MA, USA) and thermogravimetric analysis (TGA Q500) (TA Instruments, New Castle, DE, USA), as previously reported [23]. CCCA microparticles were characterized by attenuated total reflection Fourier-transform infrared spectroscopy (ATR-FTIR), recorded using an Agilent Cary 360 FTIR in the range of 4000 to 500 cm^−1^ at a resolution of 4 cm^−1^ with 32 scans on ATR (Agilent Technologies, Palo Alto, CA, USA). In addition, thermogravimetric analysis (NETZSCH TG 209 F3 Tarsus^®^, Selb, Germany) was performed, in which the samples were heated at a constant rate of 10 °C/min from room temperature to 600 °C under a nitrogen flow of 30 mL/min.

#### 3.2.5. Analysis of Synthesized Materials by Scanning Electron Microscopy (SEM)

CLPHMA hydrogels were analyzed by SEM, as previously reported [21,23], as follows: the sample was cut and loaded in the copper stub. Later, it was stained with 0.7% (*w*/*v*) phosphotungstic acid, washed, and air-dried. The sample was examined in SEM mode, in a low-voltage electron microscope (LVEM) (Delong Instruments s.r.o., Brno, Czech Republic), and was used at a nominal operating voltage of 5 kV (LVEM5).

The surface analysis of the CCCA was analyzed by SEM using an ETEC Autoscan Model U-1 scanning electron microscope coupled to a JeoL EDS device (University of Massachusetts, Worcester, MA, USA). The samples were fixed in a sample holder and covered with a gold layer for 3 min using an Edwards S150 sputter coater (BOC Edwards, São Paulo, Brazil) before analysis.

### 3.3. Capture and Desorption Assays

The compounds CFN, KCF, DMT, MTD and MTL were dissolved individually in methanol to achieve solutions of 1000 mg L^−1^. These solutions were combined in the appropriate proportions to make an aqueous model solution of 1 mg L^−1^. This solution was used as a model to determine the capture and desorption capacity of the compounds. A quantity of 30 mg of each synthesized material was used, in 10 mL of aqueous model solution. Desorption was performed in 5 mL of methanolic solution. In a first trial, the desorption was performed with mechanical agitation at 20 °C, while in a second trial, the desorption was performed in an ultrasound bath at 20 °C. An experimental design based on 2^2^ was used, with a total of 22 experiments performed for each synthesized material. The variables considered in the study were: capture time (10 to 120 min), desorption time (60 to 120 min), cryogel composition (10 wt.%, 20 wt.% and 30 wt.%, in the case of CLPHMA, and 47.3 wt.%, 52.4 wt.% and 56.5 wt.%, in the case of CCCA), and pH of the desorption solution (3, 7.3 for pure methanol, and 9). All variables were coded between −1 and 1 to have the same statistical weight. Multiple regression studies were performed to determine the statistically significant variables and the optimal experimental conditions for the desorption of the selected analytes. Finally, the synthesized materials were tested under optimal experimental conditions in a urine solution spiked with these compounds at a concentration of 1 mg L^−1^. To ensure the quality of the urine, it was sampled from a 7-year-old boy, taking the first urination of the morning, for 3 consecutive days, being refrigerated at 4 °C, until it was used in the assay.

### 3.4. Chromatographic Method

Quantitation was performed using a gas chromatograph Thermo Model TSQ DUO with Triplus injector system. The column used was RTX-5ms with the following temperature conditions: the injector temperature was 260 °C, and initially the temperature was set at 50 °C (held for 1 min), a 25 °C min^−1^ rate to 125 °C, then a 10 °C min^−1^ rate to 250 °C for 5 min holding time. Helium (99.999% purity) was used as carrier gas with a constant flow rate of 1.0 mL min^−1^. MS parameters such as the transfer line temperature (250 °C) and ion source temperature (300 °C) were maintained. Samples were ionized in MS by the positive electron impact (EI) mode using electron energy of −70 eV. The solvent delay was fixed at 3 min. The SIM mode was selected. Masses used for quantification were 164 *m*/*z* (CFN), 178 *m*/*z* (KCF), 229 *m*/*z* (DMT), 141 *m*/*z* (MTD) and 88 *m*/*z* (MTL). The total time program was 21.5 min.

## 4. Results and Discussion

### 4.1. Molecular Dynamics Simulation Results

**Figure 1:**
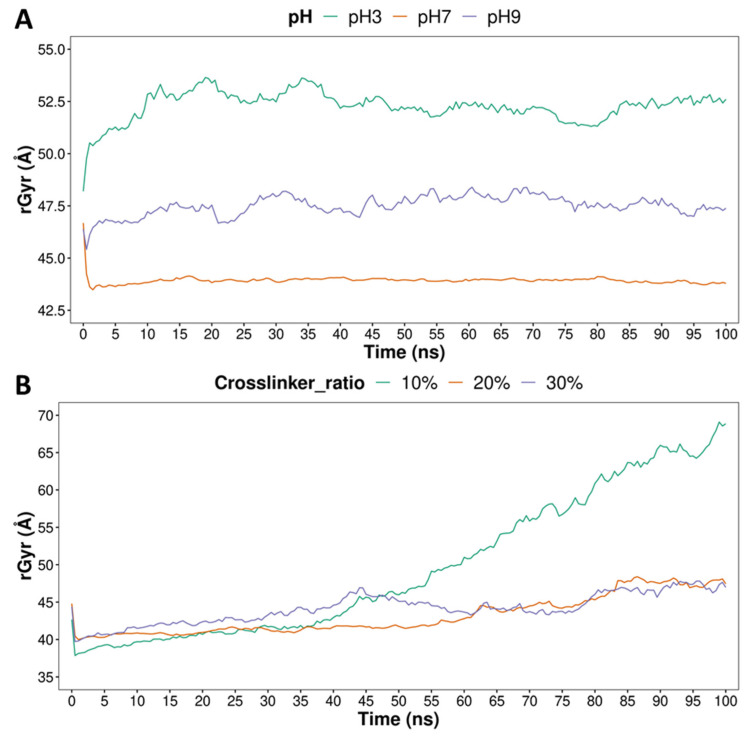
Radius of gyration (rGyr) of ALG-CT (**A**) and PVA-MA (**B**) hydrogels during the 100 ns of molecular dynamics simulation. A total of 3 pH values were considered for ALG-CT systems, and 3 crosslinker ratios were considered for PVA-MA systems.

Molecular dynamics simulations were carried out to characterize the structures of hydrogels at the molecular level and their ability to release pesticides. It is worth mentioning that for the ALG-CT hydrogel, three different simulations were run, varying the pH of the system between 3, 7 and 9. For the PVA-MA hydrogel, the proportion of MA crosslinker was varied between 10%, 20% and 30%. Each system was built with ten molecules of each pesticide inside to evaluate release in an environment solvated with methanol, mimicking the experimental assays. Figure 1 presents the calculated radius of gyration values in order to provide a structural measure of the degree of compaction of the hydrogels during the entire simulation time. In ALG-CT (Figure 1A) noticeable differences can be observed between the different pH values. The hydrogel with the largest radius of gyration is the one that was subjected to pH 3, then pH 9 and finally pH 7, with approximate values of 53 Å, 47 Å and 44 Å, respectively. In the case of PVA-MA (Figure 1B), a similar trend can be observed among hydrogels during the first half of the simulation; however, after this time, the system with a 10% crosslinker shows a noticeable increase in its radius of gyration, reaching approximately 68 Å. This does not happen with the other two crosslinker ratios that maintain their radius of gyration below 45 Å.

**Figure 2:**
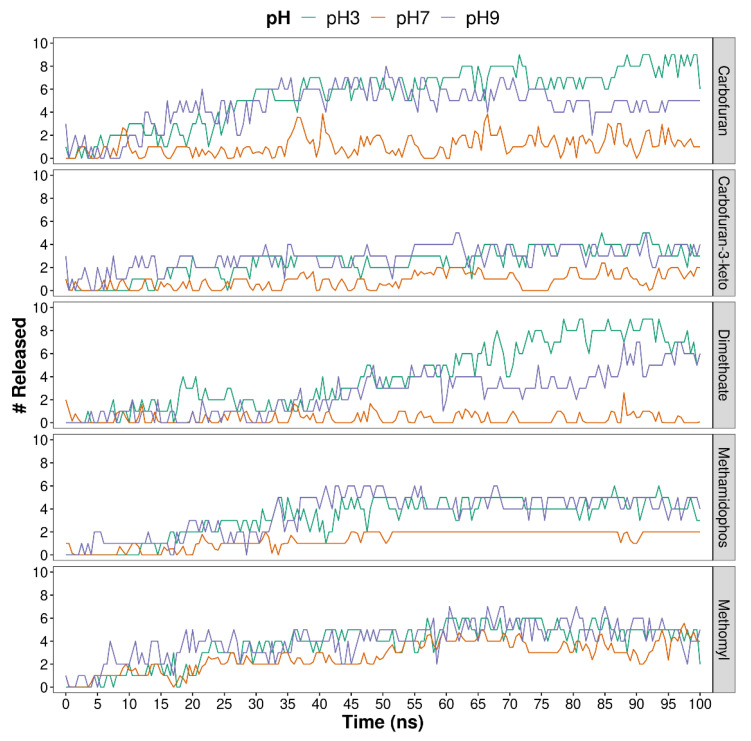
Number of compounds released from ALG-CT hydrogels, during the 100 ns of molecular dynamics simulations. A total of 3 pH values were considered. The release of pesticides was calculated during the simulation by counting, in each step, the pesticide molecules located more than 5 Å away from the hydrogel.

**Figure 3:**
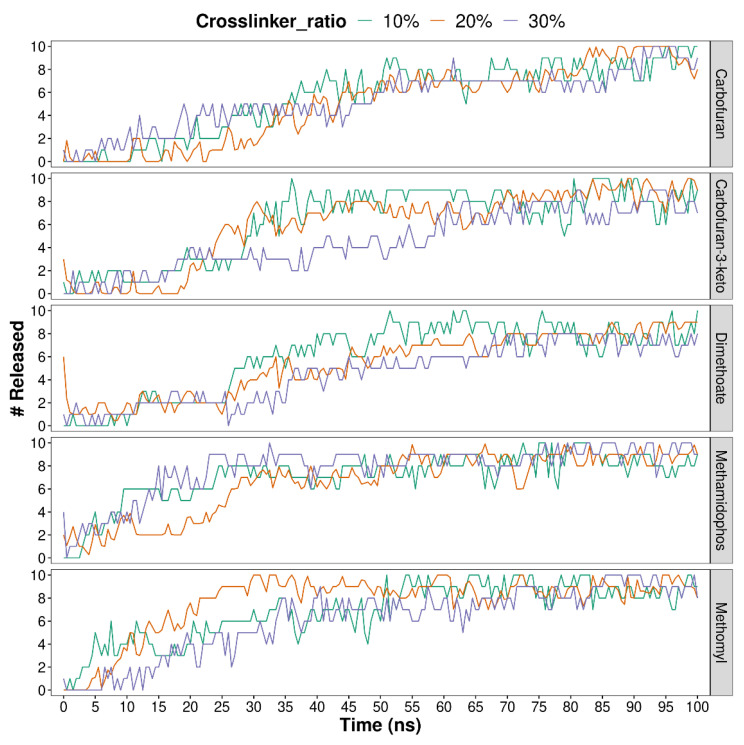
Number of pesticides released from PVA-MA hydrogels, during the 100 ns of molecular dynamics simulations. A total of 3 crosslinker ratios were considered. The release of pesticides was calculated during the simulation by counting, in each step, the pesticide molecules located more than 5 Å away from the hydrogel.

Figure 2 and Figure 3 show the release of compounds, calculated as the number of compound molecules located more than 5 Å away from the hydrogels. In the case of ALG-CT configuration (Figure 2), pesticides are released in different amounts depending on the pH. For the five compounds analyzed, the trend is that there is less release at neutral pH. Regarding pH 3 and 9, there does not seem to be a noticeable difference, except in the pesticides CFN and DMT where a greater release is observed at acidic pH.

In PVA-MA hydrogels (Figure 3) it is difficult to observe the differences between crosslinker ratios. However, it is possible to note that in most cases a complete release of pesticides is achieved.

A more detailed analysis was performed to find release differences between compounds. For this, the averages of the molecules released during the simulation and additionally the diffusion coefficients of each compound were calculated.

Table 1 shows the release and diffusion coefficients of each pesticide in the ALG-CT systems under different pH values. Additionally, the general release values and diffusion coefficients for each system are presented. Regarding the individual release of pesticides, a lower release of KCF and DMT can be observed, which also have low diffusion coefficients. On the other hand, MTL is the pesticide with the highest release at pH 7 and also the one with the highest diffusion coefficient in this same system. Considering the release of all pesticides, the lowest release at pH 7 is evident, where during the simulation the average of molecules released was 1.3 out of a total of 10. Likewise, the diffusion coefficient of all pesticides at pH 7 is the lowest compared with the other systems. Regarding the acidic and basic pH, there is no significant difference in the release, but there is in the diffusion coefficients, where at pH 9 it is 59.2 Å/ns and pH 3 only 32.9 Å/ns.

Table 2 shows the release and diffusion coefficients of each pesticide in the PVA-MA systems using the different crosslinker ratios. In addition, the general values per system are presented. It is observed that one of the pesticides with the highest release and diffusion coefficients is MTL; specifically, in the system with 20% crosslinker it presents the highest values. In the system with 30% crosslinker it has the second-highest release value and the highest diffusion coefficient. Considering the compounds with less release, KCF and DMT stand out again, which have the lowest release values and diffusion coefficients in the system with 30% crosslinker. Likewise, both pesticides have the lowest diffusion coefficients in the system with 20% crosslinker. In the general comparison of the systems, there is no clear difference between the 20% and 30% crosslinker systems; however, it is noticeable that PVA-MA with 10% crosslinker is the system with the highest release, and the one in which pesticides have the highest average diffusion coefficient (102.4 Å/ns).

**Figure 4:**
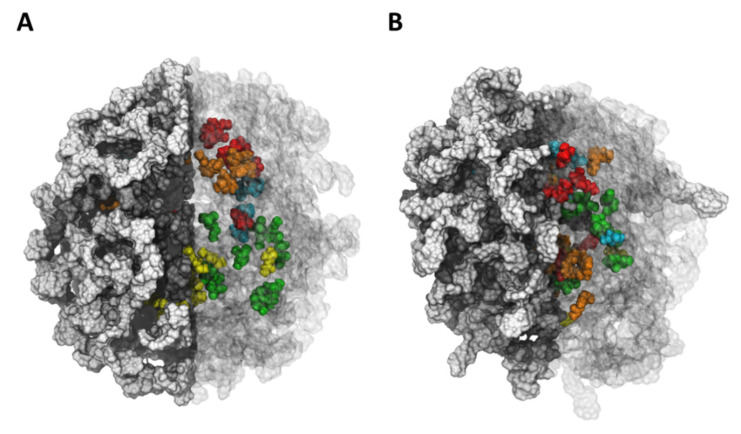
The nanoparticle shape of ALG-CT (at pH 7) at the (**A**) beginning (0 ns) and (**B**) final simulation time (100 ns). The compounds are shown in different colors. The ALG and CT chains are represented in gray and white, respectively. The particle was divided for its best representation. The left half shows an opaque representation, while the right half shows the transparent one to be able to visualize the compounds within the particle.

ALG-CT at pH 7 showed a more rigid and compact structure (Figure 4), unlike ALG-CT at pH 3 and 9, which revealed a more expanded structure. Our results suggest when the pH is different from neutral, the hydrogel loses stability due to the lower number of salt bridges between the ALG and the CT, causing a greater release of pesticides. This difference is due to the presence of salt bridges between the positively charged amino groups of CT and the negatively charged carboxylic acids of ALG. This type of interaction has already been reported previously as a determining factor in the stability of this type of formulation [35,36].

**Figure 5:**
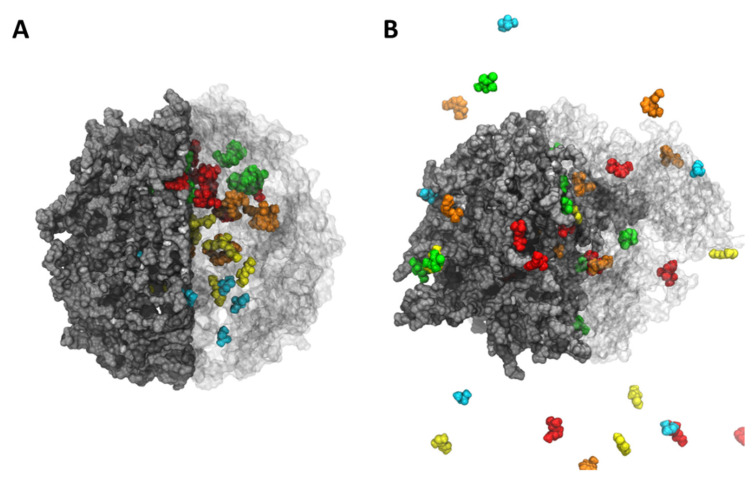
The nanoparticle shape of PVA-MA (with 30% crosslinker ratio) at the (**A**) beginning (0 ns) and (**B**) final simulation time (100 ns). The compounds are shown in different colors. The PVA-MA is represented in gray. The particle was divided for its best representation. The left half shows an opaque representation, while the right half shows the transparent one to be able to visualize the compounds within the particle.

For PVA-MA, the amount of crosslinker is relevant in the release of pesticides. Although there is no clear difference between the rates of 20% and 30%, the rate of 10% shows differences with the previous ones, being the one that reaches the highest release values and also because it has the largest radius of gyration towards the end of the simulation. Thus, our results suggest that the greater the amount of crosslinker, the more compact the structure taken on by the PVA-MA hydrogel (Figure 5), which allows fewer pesticides to be released.

Finally, the rapid release of compounds from both hydrogels occurs when they adopt a more widespread conformation. Additionally, it is noteworthy that mechanical forces such as agitation or the use of ultrasound can be key to achieving higher release rates in these type of systems.

### 4.2. Swelling Study of CLPHMA and CCCA

**Figure 6:**
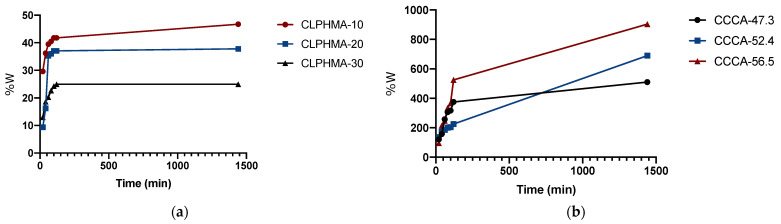
Swelling index of (**a**) CLPHMA and (**b**) CCCA in distilled water (pH 5.5) with respect to time.

The experiments were carried out with the objective of evaluating the swelling capacity of the prepared cryogels, in water, at room temperature. The swelling index for the different materials synthesized is shown in Figure 6.

This figure shows that the swelling index increases with time for all the synthesized materials. In the case of CLPHMAs, the swelling index increases rapidly in the early part, and more slowly thereafter. This behavior is due to the fact that CLPHMAs reach a maximum when swelling becomes constant. The CLPHMA-10, CLPHMA-20 and CLPHMA-30 reached the swelling equilibrium at about 100 min (see Figure 6a). A higher swelling ratio was observed for CLPHMA-10, which can be justified by the lower crosslinking density and lower rigidity. At 100 min CLPHMA-10 showed a value of 41.78%, CLPHMA-20 presented a value of 37.05%, while CLPHMA-30 presented a value of 24.92%. With respect to CCCA microparticles, they had a higher swelling ratio, reaching a swelling at 100 min of 316.67%, 204.69% and 367.86%, for CCCA-47.3, CCCA-52.4 and CCCA-56.5, respectively. The prepared CCCAs continue to swell beyond the time used in the swelling study, although their swelling rate decreases over time.

The swelling index varies depending on the nature of the polymer, the stiffness of the polymer chain, the average molecular weight, the degree of crosslinking, and the pore size of the polymeric mesh, as well as on external conditions, such as temperature and pH [23].

Considering the time in which CLPHMAs reached the swelling equilibrium, the high swelling rate of CCCAs and previous pesticide capture studies [21,23], a time range for the capture of CFN, KCF, DMT, MTD and MTL from the aqueous model solution was selected of between 10 and 120 min.

### 4.3. CT, NaALG and CCCA ATR-FTIR and TGA Analysis

**Figure 7:**
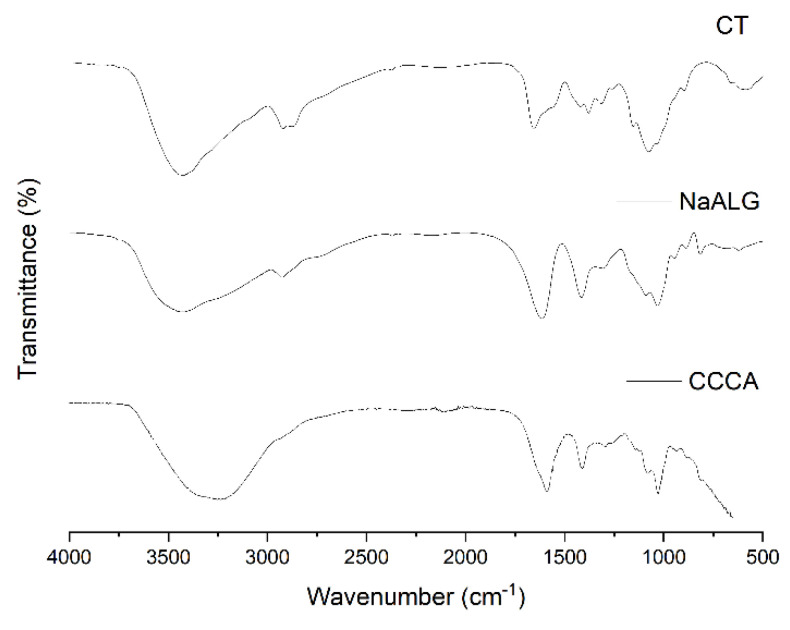
ATR-FTIR of CT, NaALG and CCCA.

ATR-FTIR is of importance to study the chemical interactions between CT and ALG occurring in the obtained microparticles. Figure 7 shows the infrared spectra for CT, NaALG, and CCCA in the wavelength range of 4000–500 cm^−1^. Analyzing the NaALG and CT infrared spectra, the common bands corresponding to the functional groups existing in their structures can be recognized. In the NaALG spectra, the widest and most intense bands found in the region 3454–3202 cm^−1^ are assigned to OH stretching vibration. In addition, the bands at 1614, 1415 and 1031 cm^−1^, were assigned to the C=O, COO^−^ and C-O groups, respectively. Finally, the characteristic peak of sodium alginate appeared at 815 cm^−1^ (Na-O).

On the other hand, the main CT bands were found at 3425, 2922 and 2876, 1655 and 1318, 1590 and 1155 cm^−1^, which can be attributed to the stretching vibration of OH, CH, NH (amine I and II), primary amino -NH_2_ a and COC groups, respectively. The band positions were within the range reported in the literature for these functional groups for both polysaccharides [37,38].

The spectra of the CCCA are also presented in Figure 7. This spectrum was characterized by the presence of absorption bands typical of the pure components; however, the CCCA spectrum, in a contrast to that of CT and NaALG, shows fewer absorption bands. For example, the band at 1570 cm^−1^, assigned to the N-H bending vibration of chitosan and the asymmetric and symmetric -C-O stretching at 1407 cm^−1^ found in the NaAlg spectra, disappeared. These results indicate that the ammonium groups (-NH_3_^+^) of CT reacted with the carboxylic groups (-COO^−^) of ALG through electrostatic interactions to form the polyelectrolyte complex (CCCA) [39]. Another notable difference was found in the band that appears in the region 3554–3100 cm^−1^—a broadening of the band attributable to the hydrogen bonds between CT and NaALG was observed.

**Figure 8:**
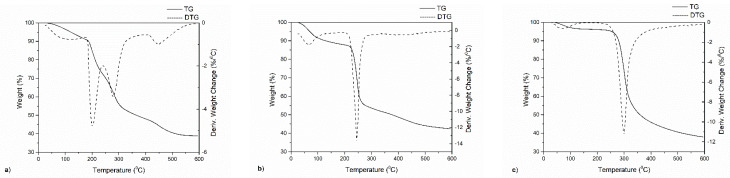
TG-DTG thermograms of CCCA (**a**), NaALG (**b**) and CT (**c**).

The thermal decomposition process of CCCA and their pure counterparts NaALG and CT were assessed to verify the influence of the structure and composition in the interval from 30 to 600 °C. The weight loss (TG) and derivate (DTG) curves for CT, NaALG, and CCCA were shown in Figure 8a–c.

The obtained results for NaALG and CT showed two degradation stages, which were reflected as two peaks on the DTG curve. The overall decomposition process of around 58% and 62% weight loss for NaALG and CT consisted of minor dehydration followed by degradation into Na_2_CO_3_ and degradation of the pyranose ring followed by the carbonized material, respectively. These weight loss processes, with peaks centered at 67.3 and 246.1 °C for the NaALG and at 62.4 and 299.1 °C for CT, were shown in Figure 8b,c, respectively. Finally, these results are in agreement with the data reported by Valdés et al. [40] and Zawadzki et al. [41] for NaALG and CT, respectively.

On the other hand, thermal degradation of CCCA was shown in Figure 8a. Analyzing the CCCA thermograms, it can be noted that the CCCA showed four-step weight loss processes, with peaks centered at 61.3, 200.5, 278.9 and 450.9 °C. These first peaks were attributed to the loss of mass through vaporization of volatile components, such as free water, present. The peak of the greatest weight loss (second step) for CCCA was significantly shifted to the lower temperatures compared with that for NaAlg. This result may be explained by the water bonded to the functional groups of both polymers, which was not completely removed in the first step of dehydration, and the degradation of carboxylic groups present in the NaALG structure. The third weight loss was characteristic of the deacetylation and partial depolymerization of the chitosan chain. This behavior was observed in a pure chitosan sample. Finally, the last degradation that occurred in the temperature range 420–500 °C may be associated with the decomposition of the polyelectrolyte complex. This fact may be considered proof of the successful formation of CCCA and agrees with the obtained results by ATR-FTIR.

### 4.4. CCCA Microparticles SEM Analysis

**Figure 9:**
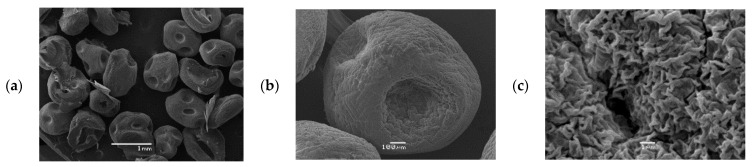
SEM pictures of CCCA microparticles dried in air, with different magnification (**a**) 1 mm, (**b**) 100 µm, and (**c**) 1 µm.

Scanning electron micrographs of dry CCCA microparticles and their surface morphology are illustrated in Figure 9 at different scales. Analyzing Figure 9a, an acceptable spherical shape and a diameter of approximately 1.00 mm can be seen for the CCCA microparticles. It is important to clarify that the disk shape shown in their figure was due to air drying. In addition, the CCCAs show a soft surface (See Figure 9b). Finally, Figure 9c shows that another notable feature of the microcapsule surface was its high porosity and rough surface marked by large wrinkles.

### 4.5. Capture and Desorption Assays

Table 3 and Table 4 present the results obtained for the desorption tests of CFN, KCF, DMT, MTD and MTL from aqueous solutions, using mechanical agitation in the desorption process, and using CLPHMA (Table 3) and CCCA (Table 4) as sorbent phases. To eliminate the errors associated with the different masses of the materials used, the results were expressed as the released concentration of the compounds under study per mg of sorbent material.

It is observed that in most of the experiments carried out, CCCA microparticles release a higher concentration of the compounds studied per mg of CCCA than in the case of CLPHMAs, regardless of the type of agitation used in the desorption process.

Considering 30 mg of sorbent phase, and given that each compound is at a concentration of 1 mg/L in the model aqueous solution, for the desorption process to be quantitative, 0.033 mg/L of compound should be released per mg of material. Thus, it is observed that mechanical agitation is not sufficient for the quantitative desorption of the compounds, reaching a maximum desorption of 10% of MTD, when using CLPHMA as sorbent phase, and a maximum of 20% in the case of CFN when using CCCA.

Tables S1 and S2 present the results obtained for the desorption tests of CFN, KCF, DMT, MTD and MTL from aqueous solutions, using an ultrasonic bath at 20 °C in the desorption process, with CLPHMA (Table S1) and CCCA (Table S2) as sorbent phases. Ultrasonic agitation has been used successfully in the release of compounds from hydrogels [14]. The use of ultrasound promotes the implosion of cavitational bubbles, the formation of microjets, microturbulence, high velocity interparticle collisions and the perturbation in microporous particles results in an enhanced extraction and accelerated chemical reactions [42].

When comparing the systems where CPLHMA is used, some increases in the desorption of compounds are observed when switching from mechanical agitation to ultrasound, as in the case of CFN (experiments 4, 9, 14 and 15), KCF (experiments 1, 5 and 11), DMT (experiments 1, 2, 7 and 20) and MTD (experiments 2, 4, 9, 14–18, 20 and 21), and in the case of MTL, mechanical agitation has a better response. In general, there is no trend that implies a correlation between the type of agitation and the release of the compounds under study.

In the case of systems using CCCA, it is observed that in some cases such as CFN (experiments 18 and 20), KCF (experiments 2, 3, 6, 9, 10, 12–15, 19 and 20), DMT (experiments 8, 12, 18–21), MTD (experiments 5, 10, 11, 14, 15, 18–22) and MTL (experiments 3, 4, 6, 9–15, 19–21) ultrasonic agitation is more efficient for the release of these compounds.

In general terms, no clear trends in the release of the compounds under study were observed, so a multiple regression analysis was performed. Table 5 and Table 6 present the results for the desorption tests of the compounds under study, using CLPHMA and CCCA as sorbent material, with mechanical agitation. It is observed that for all the analytes under study the *p*-value in the ANOVA is greater than 0.05, so there is not a statistically significant relationship between the variables at the 95.0% or higher confidence level.

Tables S3 and S4 present the results for the desorption tests of the compounds under study, using CLPHMA and CCCA as sorbent material, with ultrasound agitation. It is observed that for all the analytes under study the *p*-value in the ANOVA is greater than 0.05, so there is not a statistically significant relationship between the variables at the 95.0% or higher confidence level.

Considering the significance of each experimental variable studied, from the multiple regression studies, we proceeded to formulate a series of experiments described in Table 7. These experiments were carried out in urine spiked with the compounds studied at 1 mg/L, to see their recovery, in triplicate (Table 8). The pH of the urine sample was 5.8, so the extractability conditions should not vary greatly with respect to the model aqueous solution in this parameter.

It is observed that the best results in the case of CFN, KCF and MTL (Experiment 1: capture time, 120 min; desorption time, 120 min; sorbent, CLPHMA-30; pH 9, and mechanical agitation). In the case of DMT, the best experimental conditions were from experiment 3: capture time, 120 min; desorption time, 120 min; sorbent, CCCA-56.5; pH 3, and mechanical agitation. In the case of MTD, the best extraction conditions were from experiment 4: capture time, 10 min; desorption time, 60 min; sorbent, CCCA-47.3; pH 3, and mechanical agitation. Under these experimental conditions, the maximum recoveries were of the order of 11% (CFN), 3% (KCF), 53% (DMT), 18% (MTD) and 35% (MTL). Regarding the pH conditions and the material used as sorbent phase, in the case of CLPHMA, pH variations do not greatly influence the structure at the molecular level, while in the case of CCCA, at acid pH, the particle expands and loses stability, releasing the compounds in a better way.

In the experiments where ultrasonic agitation was used in the desorption process, contrary to expectations, the recoveries were lower than using mechanical agitation. In Experiment 6, the capture time may have been insufficient for CLPHMA to extract CFN, DMT, MTD and MTL compounds from urine. In Experiment 7, the desorption time may have affected the release capacity of CCCA with respect to KCF. In addition, the pH 9 of the desorption solution makes the stability of CCCA higher than at acidic pH, so the desorption process may be less effective.

## 5. Conclusions

MDS is a very powerful tool for predicting the conditions under which the synthesized materials can release the compounds under study. Its limitations lie in the time in which these simulations are performed; it is not possible to predict how the capture and desorption time influences the studied systems.

In the experimental assay, it was observed that in the release of certain compounds, the pH was not statistically significant, mainly in the PVA-MA systems. According to the theoretical predictions of the pKa in the PVA monomer and in the MA crosslinker, these do not undergo changes in the protonation states of the hydroxyl groups in the pH ranges studied. Therefore, pH does not directly affect the structural conformation of PVA-MA, thus explaining the low significance of pH in the release of pesticides for this hydrogel. Unlike PVA-MA, the ALG-CT microparticles are significantly affected by pH in the release of pesticides. This was observed at an experimental and theoretical level and is due to the changes in the protonation states suffered by the monomers of ALG and CHI at different pH. These changes ultimately affect the stability and compaction of the hydrogel, which is key to the release of pesticides.

The statistical analysis of the results allows us to correlate the obtained results in a better way, as well as to determine the statistical significance of the experimental variables considered in the study, allowing the simplification of the obtained models. It is also a tool that complements MDS, from the experimental point of view.

Despite the low recoveries obtained at the experimental level, the materials used look promising as new sorbent phases for the extraction of pesticides and their metabolites from urine, and their potential applications include their use in modern and validated solid–liquid extraction systems, such as stir bar sorptive extraction and rotating disk solvent extraction. However, further experimental trials are needed to improve the desorption of the compounds, as well as MDS studies to broaden the range of compounds that can be quantitatively desorbed.

## Figures and Tables

**Table 1:** Release and diffusion coefficients (D.C.) of selected compounds in ALG-CT systems at different pH values. The release of pesticides was calculated during the simulation by counting, in each step, the pesticide molecules located more than 5 Å away from the hydrogel. The release values (# Release and # Release per Hydrogel) correspond to the average of all the steps.

System	Compound	# Release	D.C. (Å/ns)	# Release per Hydrogel	D.C. (Å/ns) per Hydrogel
ALG-CT at pH 9	CFN	4.53	96.6	3.5	59.2
	KCF	2.87	19.1		
	DMT	2.60	40.4		
	MTD R	3.58	86.6		
	MTD S		66.5		
	MTL	4.07	46.3		
ALG-CT at pH 7	CFN	1.1	1.5	1.3	7.8
	KCF	0.8	8.8		
	DMT	0.4	0.5		
	MTD R	1.4	6.6		
	MTD S		4.6		
	MTL	2.7	24.9		
ALG-CT at pH 3	CFN	5.5	40.3	3.8	32.9
	KCF	2.5	28.2		
	DMT	4.1	22.5		
	MTD R	3.3	2.2		
	MTD S		41.3		
	MTL	3.7	63.2		

**Table 2:** Release and diffusion coefficients (D.C.) of selected compounds in PVA-MA systems with different crosslinker ratios. The release of pesticides was calculated during the simulation by counting, in each step, the pesticide molecules located more than 5 Å away from the hydrogel. The release values (# Release and # Release per Hydrogel) correspond to the average of all the steps.

Hydrogel	Compound	# Release	D.C. (Å/ns)	# Release per Hydrogel	D.C (Å/ns) per Hydrogel
PVA-MA with 10% crosslinker	CFN	5.6	74.3	6.4	102.4
	KCF	6.3	66.5		
	DMT	6.0	120.6		
	MTD R	7.1	146.7		
	MTD S		64.7		
	MTL	6.9	141.5		
PVA-MA with 20% crosslinker	CFN	5.1	57.2	6.1	78.8
	KCF	6.0	47.5		
	DMT	5.4	55.1		
	MTD R	6.6	60.1		
	MTD S		80.6		
	MTL	7.6	172.4		
PVA-MA with 30% crosslinker	CFN	5.4	76.3	5.7	85.4
	KCF	4.7	41.2		
	DMT	4.7	72.6		
	MTD R	7.8	108.4		
	MTD S		95.8		
	MTL	6.1	118.0		

**Table 3:** Assay to describe and optimize the capture and desorption of selected compounds by CLPHMA with mechanical agitation. Numbers in parentheses correspond to the coded values of the variables under study (ND: Not Detected).

Exp	Capture Time (min)	Desorption Time (min)	CLPHMA Composition (%)	pH of Desorption Solution (-)	mg/L of Compound Released per mg of CLPHMA				
					CFN	KCF	DMT	MTD	MTL
1	120 (1)	60 (−1)	30 (1)	3 (−1)	0.0005	ND	ND	ND	0.0004
2	10 (−1)	120 (1)	10 (−1)	9 (1)	0.0009	ND	ND	ND	ND
3	120 (1)	60 (−1)	30 (1)	3 (−1)	0.0010	0.0014	ND	0.0024	ND
4	120 (1)	60 (−1)	30 (1)	3 (−1)	0.0009	ND	0.0015	ND	0.0004
5	120 (1)	120 (1)	30 (1)	9 (1)	0.0005	ND	ND	ND	ND
6	10 (−1)	60 (−1)	10 (−1)	3 (−1)	0.0005	ND	ND	0.0014	0.0004
7	120 (1)	120 (1)	30 (1)	9 (1)	0.0007	0.0009	ND	ND	ND
8	10 (−1)	120 (1)	10 (−1)	9 (1)	0.0010	ND	ND	ND	ND
9	10 (−1)	120 (1)	10 (−1)	9 (1)	0.0006	0.0010	ND	0.0022	0.0007
10	10 (−1)	120 (1)	10 (−1)	9 (1)	0.0007	ND	ND	ND	0.0003
11	120 (1)	60 (−1)	30 (1)	3 (−1)	0.0005	ND	0.0011	ND	ND
12	120 (1)	120 (1)	30 (1)	9 (1)	0.0008	0.0008	ND	ND	ND
13	10 (−1)	60 (−1)	10 (−1)	3 (−1)	0.0009	0.0008	ND	ND	ND
14	10 (−1)	60 (−1)	10 (−1)	3 (−1)	ND	ND	ND	ND	ND
15	10 (−1)	60 (−1)	10 (−1)	3 (−1)	ND	ND	ND	ND	ND
16	120 (1)	120 (1)	30 (1)	9 (1)	0.0008	ND	ND	ND	ND
17	10 (−1)	90 (0)	10 (−1)	7.3 (0.43)	0.0005	ND	ND	ND	ND
18	65 (0)	90 (0)	20 (0)	7.3 (0.43)	0.0005	ND	0.0014	ND	ND
19	120 (1)	60 (−1)	30 (1)	7.3 (0.43)	0.0021	ND	ND	ND	0.0005
20	10 (−1)	60 (−1)	20 (0)	7.3 (0.43)	0.0008	ND	0.0012	ND	ND
21	65 (0)	120 (1)	30 (1)	7.3 (0.43)	0.0006	ND	ND	ND	ND
22	120 (1)	120 (1)	10 (−1)	7.3 (0.43)	0.0012	ND	ND	0.0031	0.0004

**Table 4:** Assay to describe and optimize the capture and desorption of selected compounds by CCCA microparticles with mechanical agitation. Numbers in parentheses correspond to the coded values of the variables under study (ND: Not Detected).

Exp	Capture Time (min)	Desorption Time (min)	CCCA Composition (%)	pH of Desorption Solution (-)	mg/L of Compound Released per mg of CCCA				
					CFN	KCF	DMT	MTD	MTL
1	120 (1)	60 (−1)	56.5 (1)	3 (−1)	0.0065	0.0132	0.0088	0.0095	0.0253
2	10 (−1)	120 (1)	47.3 (−1)	9 (1)	0.0033	0.0030	0.0043	0.0141	0.0029
3	120 (1)	60 (−1)	56.5 (1)	3 (−1)	0.0031	0.0020	0.0012	0.0087	0.0023
4	120 (1)	60 (−1)	56.5 (1)	3 (−1)	0.0035	0.0025	0.0060	0.0142	0.0019
5	120 (1)	120 (1)	56.5 (1)	9 (1)	0.0034	0.0052	0.0015	0.0019	0.0072
6	10 (−1)	60 (−1)	47.3 (−1)	3 (−1)	0.0017	0.0010	0.0058	0.0051	0.0010
7	120 (1)	120 (1)	56.5 (1)	9 (1)	0.0026	0.0052	0.0019	0.0051	0.0061
8	10 (−1)	120 (1)	47.3 (−1)	9 (1)	0.0019	0.0016	0.0017	0.0073	0.0020
9	10 (−1)	120 (1)	47.3 (−1)	9 (1)	0.0010	0.0011	0.0020	0.0034	ND
10	10 (−1)	120 (1)	47.3 (−1)	9 (1)	0.0010	0.0008	0.0034	0.0023	0.0006
11	120 (1)	60 (−1)	56.5 (1)	3 (−1)	0.0017	0.0026	0.0035	0.0017	ND
12	120 (1)	120 (1)	56.5 (1)	9 (1)	0.0022	0.0011	0.0011	0.0078	0.0006
13	10 (−1)	60 (−1)	47.3 (−1)	3 (−1)	0.0026	0.0008	0.0271	0.0087	ND
14	10 (−1)	60 (−1)	47.3 (−1)	3 (−1)	0.0011	0.0013	0.0017	ND	0.0003
15	10 (−1)	60 (−1)	47.3 (−1)	3 (−1)	0.0007	0.0008	ND	0.0022	ND
16	120 (1)	120 (1)	56.5 (1)	9 (1)	0.0018	0.0023	ND	0.0025	0.0004
17	10 (−1)	90 (0)	47.3 (−1)	7.3 (0.43)	0.0014	0.0013	ND	0.0035	0.0009
18	65 (0)	90 (0)	52.4 (0.11)	7.3 (0.43)	0.0018	0.0018	ND	ND	0.0016
19	120 (1)	60 (−1)	56.5 (1)	7.3 (0.43)	0.0014	0.0014	ND	0.0032	ND
20	10 (−1)	60 (−1)	52.4 (0.11)	7.3 (0.43)	0.0005	ND	0.0012	0.0040	ND
21	65 (0)	120 (1)	56.5 (1)	7.3 (0.43)	0.0017	0.0017	ND	0.0017	0.0004
22	120 (1)	120 (1)	47.3 (−1)	7.3 (0.43)	0.0018	0.0026	0.0016	0.0031	0.0034

**Table 5:** Summary of the multifactorial multiple regression analysis for the analytes under study and their experimental variables, using CLPHMA, and mechanical agitation for extraction.

Analyte	Influence				ANOVA Model *p*-Value
	A	B	C	D	
CFN	+	-	- (NSS)	+	0.0654
	Equation of the fitted model			R^2^ (%)	
	CFN = 0.000711575 + 0.000391122 × A − 0.000462556 × B + 0.000575588 × D			38.90	
KCF	+	+	+	- (NSS)	0.9774
	Equation of the fitted model			R^2^ (%)	
	KCF = 0.00022443 + 0.0000213213 × A + 0.0000400718 × B + 0.0000480358 × C			2.52	
DMT	- (NSS)	-	+	+	0.4353
	Equation of the fitted model			R^2^ (%)	
	DMT = 0.000229189 − 0.00026971 × B + 0.000158496 × C + 0.000119232 × D			19.03	
MTD	+	+ (NSS)	-	-	0.0752
	Equation of the fitted model			R^2^ (%)	
	MTD = 0.000420364 + 0.000906789 × A − 0.000964469 × C − 0.000382322 × D			37.72	
MTL	+	-	-	+ (NSS)	0.5568
	Equation of the fitted model			R^2^ (%)	
	MTL = 0.000137202 + 0.000168035 × A − 0.000107823 × B − 0.000175881 × C			15.41	

A: capture time; B: desorption time; C: hydrogel composition; and D: pH of desorption solution (NSS: not statistically significant at the 95.0% or higher confidence level)

**Table 6:** Summary of the multifactorial multiple regression analysis for the analytes under study and their experimental variables, using CCCA, and mechanical agitation for extraction.

Analyte	Influence				ANOVA Model *p*-Value
	A	B	C	D	
CFN	+	+	+ (NSS)	-	0.2094
	Equation of the fitted model			R^2^ (%)	
	CFN = 0.00213976 + 0.00043176 × A + 0.000449696 × B − 0.000705095 × D			27.88	
KCF	+	+	+ (NSS)	-	0.2928
	Equation of the fitted model			R^2^ (%)	
	KCF = 0.00244117 + 0.00104561 × A + 0.000665528 × B − 0.000944831 × D			24.09	
DMT	-	+ (NSS)	-	-	0.4593
	Equation of the fitted model			R^2^ (%)	
	DMT = 0.00339492 − 0.000318947 × A − 0.000913688 × C − 0.00281292 × D			18.28	
MTD	+	-	-	- (NSS)	0.9874
	Equation of the fitted model			R^2^ (%)	
	MTD = 0.0050002 + 0.00050246 × A − 0.000409299 × B − 0.000141824 × C − 0.0000353008 × D			1.85	
MTL	+	+	+ (NSS)	-	0.5987
	Equation of the fitted model			R^2^ (%)	
	MTL = 0.00262437 + 0.00182125 × A + 0.000737128 × B − 0.00146819 × D			14.25	

A: capture time; B: desorption time; C: microparticle composition; and D: pH of desorption solution (NSS: not statistically significant at the 95.0% or higher confidence level)

**Table 7:** Experiments to test the release of the compounds studied by the synthesized materials in urine samples. Numbers in parentheses correspond to the coded values of the variables under study.

Experiment (n = 3)	Sorbent Type and Agitation	Capture Time (min)	Desorption Time (min)	Sorbent Composition (%)	pH (-)
1	CLPHMA, Mechanical agitation	120 (1)	120 (1)	30 (1)	9 (1)
2		120 (1)	60 (−1)	10 (−1)	3 (−1)
3	CCCA, Mechanical agitation	120 (1)	120 (1)	56.5 (1)	3 (−1)
4		10 (−1)	60 (−1)	47.3 (−1)	3 (−1)
5	CLPHMA, Ultrasonic agitation	120 (1)	60 (−1)	10 (−1)	9 (1)
6		10 (−1)	120 (1)	10 (−1)	3 (−1)
7	CCCA, Ultrasonic agitation	120 (1)	60 (−1)	47.3 (−1)	9 (1)
8		10 (−1)	120 (1)	56.5 (1)	3 (−1)

**Table 8:** Results of desorption tests of the compounds under study in selected optimal experimental conditions in urine, for the different sorbent materials tested.

	mg/L of Compound Released per mg of CCCA (Mean ± SD)				
	CFN	KCF	DMT	MTD	MTL
1	0.0036 ± 0.0010	0.0035 ± 0.0005	0.0054 ± 0.0009	0.0045 ± 0.0004	0.0115 ± 0.0028
2	0.0022 ± 0.0001	0.0014 ± 0.0001	0.0089 ± 0.0003	0.0048 ± 0.0004	0.0094 ± 0.0028
3	0.0030 ± 0.0003	ND	0.0176 ± 0.0034	0.0040 ± 0.0004	ND
4	0.0024 ± 0.0001	0.0028 ± 0.0002	0.0080 ± 0.0004	0.0059 ± 0.0011	0.0094 ± 0.0008
5	ND	0.0020 ± 0.0001	0.0163 ± 0.0007	0.0050 ± 0.0003	0.0050 ± 0.0008
6	ND	0.0017 ± 0.0002	ND	ND	ND
7	0.0025 ± 0.0002	ND	0.0132 ± 0.0010	0.0057 ± 0.0004	0.0039 ± 0.0004
8	0.0018 ± 0.0001	0.0024 ± 0.0001	0.0063 ± 0.0009	0.0051 ± 0.0005	0.0084 ± 0.0004

ND: not detected

## Supplementary Materials

The following are available online at https://www.mdpi.com/article/10.3390/polym13223993/s1, Figure S1: Scheme of quantity and distribution of the MA for each substructure of PVA-MA hydrogels, Table S1: Assay to describe and optimize the capture and desorption of selected compounds by CLPHMA with ultrasonic agitation, at 20 °C. Numbers in parentheses correspond to the coded values of the variables under study (ND: not detected), Table S2: Assay to describe and optimize the capture and desorption of selected compounds by CCCA microparticles with ultrasonic agitation at 20 °C. Numbers in parentheses correspond to the coded values of the variables under study (ND: not detected), Table S3: Summary of the multifactorial multiple regression analysis for the analytes under study and their experimental variables, using CLPHMA, and ultrasonic agitation for extraction (where A: capture time; B: desorption time; C: hydrogel composition; and D: pH of desorption solution) (NSS: not statistically significant at the 95.0% or higher confidence level), Table S4: Summary of the multifactorial multiple regression analysis for the analytes under study and their experimental variables, using CCCA, and ultrasonic agitation for extraction (where A: capture time; B: desorption time; C: hydrogel composition; and D: pH of desorption solution) (NSS: not statistically significant at the 95.0% or higher confidence level).

## References

[1] Dar M.A., Kaushik G., Villarreal-Chiu J.F. (2019). Pollution status and bioremediation of chlorpyrifos in environmental matrices by the application of bacterial communities: A review. J. Environ. Manag..

[2] Fatunsin O.T., Oyeyiola A.O., Moshood M.O., Akanbi L.M., Fadahunsi D.E. (2020). Dietary risk assessment of organophosphate and carbamate pesticide residues in commonly eaten food crops. Sci. Afr..

[3] Jing Y., Krauss M., Zschieschang S., Miltner A., Butkovskyi A., Eggen T., Kastner M., Nowak K.M. (2021). Superabsorbent polymer as a supplement substrate of constructed wetland to retain pesticides from agricultural runoff. Water Res..

[4] Li G., Zhang X., Liu T., Fan H., Liu H., Li S., Wang D., Ding L. (2021). Dynamic microwave-assisted extraction combined with liquid phase microextraction based on the solidification of a floating drop for the analysis of organochlorine pesticides in grains followed by GC. Food Sci. Hum. Wellness.

[5] Cequier E., Sakhi A.K., Haug L.S., Thomsen C. (2017). Exposure to organophosphorous pesticides in Norwegian mothers and their children: Diurnal variability in concentrations of their biomarkers and associations with food consumption. Sci. Total Environ..

[6] Dervilly-Pinel G., Guérin T., Minvielle B., Travel A., Normand J., Bourin M., Royer E., Dubreil E., Mompelat S., Hommet S. (2017). Micropollutants and chemical residues in organic and conventional meat. Food Chem..

[7] Mahmoud A.F.A., Ikenaka Y., Yohannes Y.B., Darwish W.S., Eldaly E.A., Morshdy A.E.M.A., Nakayama S.M.M., Mizukawa H., Ishizuka M. (2016). Distribution and health risk assessment of organochlorine pesticides (OCPs) residue in edible cattle tissues from northeastern part of Egypt: High accumulation level of OCPs in tongue. Chemosphere.

[8] Sookhtanlou M., Allahyari M.S., Surujlal J. (2021). Health risk of potato farmers exposed to overuse of chemical pesticides in Iran. Saf. Health.

[9] Fernández-Peralbo M.A., Luque de Castro M.D. (2012). Preparation of urine samples prior to targeted or untargeted metabolomics mass-spectrometry analysis. TrAC-Trends Anal. Chem..

[10] Wang Q., Zuo Z., Cheung C.K.C., Leung S.S.Y. (2019). Updates on thermosensitive hydrogel for nasal, ocular and cutaneous delivery. Int. J. Pharm..

[11] Coukouma A.E., Asher S.A. (2018). Increased volume responsiveness of macroporous hydrogels. Sens. Actuators B Chem..

[12] Mahinroosta M., Farsangi Z.J., Allahverdi A., Shakoori Z. (2018). Hydrogels as intelligent materials: A brief review of synthesis, properties and applications. Mater. Today Chem..

[13] Wu T., Huang J., Jiang Y., Hu Y., Ye X., Liu D., Chen J. (2018). Formation of hydrogels based on chitosan/alginate for the delivery of lysozyme and their antibacterial activity. Food Chem..

[14] Jiang S., Liu S., Feng W. (2011). PVA hydrogel properties for biomedical application. J. Mech. Behav. Biomed. Mater..

[15] Meng X., Tian F., Yang J., He C.N., Xing N., Li F. (2010). Chitosan and alginate polyelectrolyte complex membranes and their properties for wound dressing application. J. Mater. Sci. Mater. Med..

[16] Paradossi G., Cavalieri F., Chiessi E., Spagnoli C., Cowman M.K. (2003). Poly(vinyl alcohol) as versatile biomaterial for potential biomedical applications. J. Mater. Sci. Mater. Med..

[17] Cabrera-Barjas G., Gallardo F., Nesic A., Taboada E., Marican A., Mirabal-Gallardo Y., Avila-Salas F., Delgado N., de Armas-Ricard M., Valdes O. (2020). Utilization of industrial by-product fungal biomass from *Aspergillus niger* and *Fusarium culmorum* to obtain biosorbents for removal of pesticide and metal ions from aqueous solutions. J. Environ. Chem. Eng..

[18] El Harmoudi H., El Gaini L., Daoudi E., Rhazi M., Boughaleb Y., El Mhammedi M.A., Migalzka-Zalas A., Bakasse M. (2014). Removal of 2,4-D from aqueous solutions by adsorption processes using two biopolymers: Chitin and chitosan and their optical properties. Opt. Mater..

[19] Etcheberry M., Cappa V., Trelles J., Zanini G. (2017). Montmorillonite-alginate beads: Natural mineral and biopolymers based sorbent of paraquat herbicides. J. Environ. Chem. Eng..

[20] Lu L.C., Wang C.I., Sye W.F. (2011). Applications of chitosan beads and porous crab shell powder for the removal of 17 organochlorine pesticides (OCPs) in water solution. Carbohydr. Polym..

[21] Avila-Salas F., Marican A., Villaseñor J., Arenas-Salinas M., Argandoña Y., Caballero J., Durán-Lara E.F. (2018). In-Silico Design, Synthesis and Evaluation of a Nanostructured Hydrogel as a Dimethoate Removal Agent. Nanomaterials.

[22] Hui B., Zhang Y., Ye L. (2014). Preparation of PVA hydrogel beads and adsorption mechanism for advanced phosphate removal. Chem. Eng. J..

[23] Valdés O., Ávila-Salas F., Marican A., Fuentealba N., Villaseñor J., Arenas-Salinas M., Argandoña Y., Durán-Lara E.F. (2018). Methamidophos removal from aqueous solutions using a super adsorbent based on crosslinked poly(vinyl alcohol) hydrogel. J. Appl. Polym. Sci..

[24] Sabitha P., Vijaya Ratna J., Ravindra Reddy K. (2010). Design and evaluation of controlled release chitosan-calcium alginate microcapsules of anti tubercular drugs for oral use. Int. J. Chemtech Res..

[25] Maestro S. (2020). Schrödinger Release 2021-1.

[26] Shelley J.C., Cholleti A., Frye L.L., Greenwood J.R., Timlin M.R., Uchimaya M. (2007). Epik: A software program for pKa prediction and protonation state generation for drug-like molecules. J. Comput. Aided. Mol. Des..

[27] Case D.A., Aktulga H.M., Belfon K., Ben-Shalom I.Y., Brozell S.R., Cerutti D.S., Cheatham T.E., Cruzeiro V.W.D., Darden T.A., Duke R.E. Amber 2021. University of California, San Francisco. https://ambermd.org/index.php.

[28] O’Boyle N.M., Banck M., James C.A., Morley C., Vandermeersch T., Hutchison G.R. (2011). Open Babel: An Open chemical toolbox. J. Cheminform..

[29] Jorgensen W.L., Tirado-Rives J. (1988). The OPLS Potential Functions for Proteins. Energy Minimizations for Crystals of Cyclic Peptides and Crambin. J. Am. Chem. Soc..

[30] Martínez L., Andrade R., Birgin E.G., Martínez J.M. (2009). PACKMOL: A package for building initial configurations for molecular dynamics simulations. J. Comput. Chem..

[31] Giorgino T. (2019). Computing diffusion coefficients in macromolecular simulations: The Diffusion Coefficient Tool for VMD. J. Open Source Softw..

[32] Schanuel F.S., Santos K.S.R., Monte-Alto-Costa A., de Oliveira M.G. (2015). Combined Nitric Oxide-releasing Poly(vinyl alcohol) Film/F127 Hydrogel for Accelerating Wound Healing. Colloids Surf. B Biointerfaces.

[33] Guastaferro M., Reverchon E., Baldino L. (2021). Polysaccaride-Based Aerogel Production for Biomedical Applications: A Comparative Review. Materials.

[34] Gonzalez-Rodrıguez M.L., Holgado M.A., Sanchez-Lafuente C., Rabasco A.M., Fini A. (2002). Alginate/chitosan particulate systems for sodium diclofenac release. Int. J. Pharm. Sci. Res..

[35] Xu Y., Zhan C., Fan L., Wang L., Zheng H. (2007). Preparation of dual crosslinked alginate–chitosan blend gel beads and in vitro controlled release in oral site-specific drug delivery system. Int. J. Pharm..

[36] Caetano L., Almeida A., Gonçalves L. (2016). Effect of Experimental Parameters on Alginate/Chitosan Microparticles for BCG Encapsulation. Mar. Drugs.

[37] Shaari N., Kamarudin S.K. (2020). Sodium alginate/alumina composite biomembrane preparation and performance in DMFC application. Polym. Test..

[38] Balau L., Lisa G., Popa M., Tura V., Melnig V. (2004). Physico-chemical properties of Chitosan films. Open Chem..

[39] Sankalia M.G., Mashru R.C., Sankalia J.M., Sotariya V.B. (2007). Reversed chitosan—Alginate polyelectrolyte complex for stability improvement of alpha-amylase: Optimization and physicochemical characterization. Eur. J. Pharm. Biopharm..

[40] Valdés O., Alexandrova L., Zaldivar D., Katime I. (2012). A comparative study of two polyelectrolyte complexes. J. Appl. Polym. Sci..

[41] Zawadzki J., Kaczmarek H. (2010). Thermal treatment of chitosan in various conditions. Carbohydr. Polym..

[42] Kadam S.U., Tiwari B.K., Álvarez C., O’Donnell C.P. (2015). Ultrasound applications for the extraction, identification and delivery of food proteins and bioactive peptides. Trends Food Sci. Technol..

