# Supplementary material for: Use of Poly(vinyl alcohol)-Malic Acid (CLHPMA) Hydrogels and Chitosan Coated Calcium Alginate (CCCA) Microparticles as Potential Sorbent Phases for the Extraction and Quantitative Determination of Pesticides from Aqueous Solutions
Source: Polymers (Basel). 2021 Nov 19;13(22):3993. doi: 10.3390/polym13223993 (PMC8619381; doi:10.3390/polym13223993)
Supplement: Supplementary file 1 [file polymers-13-03993-s001.zip › polymers-1465075-supplementary.pdf]

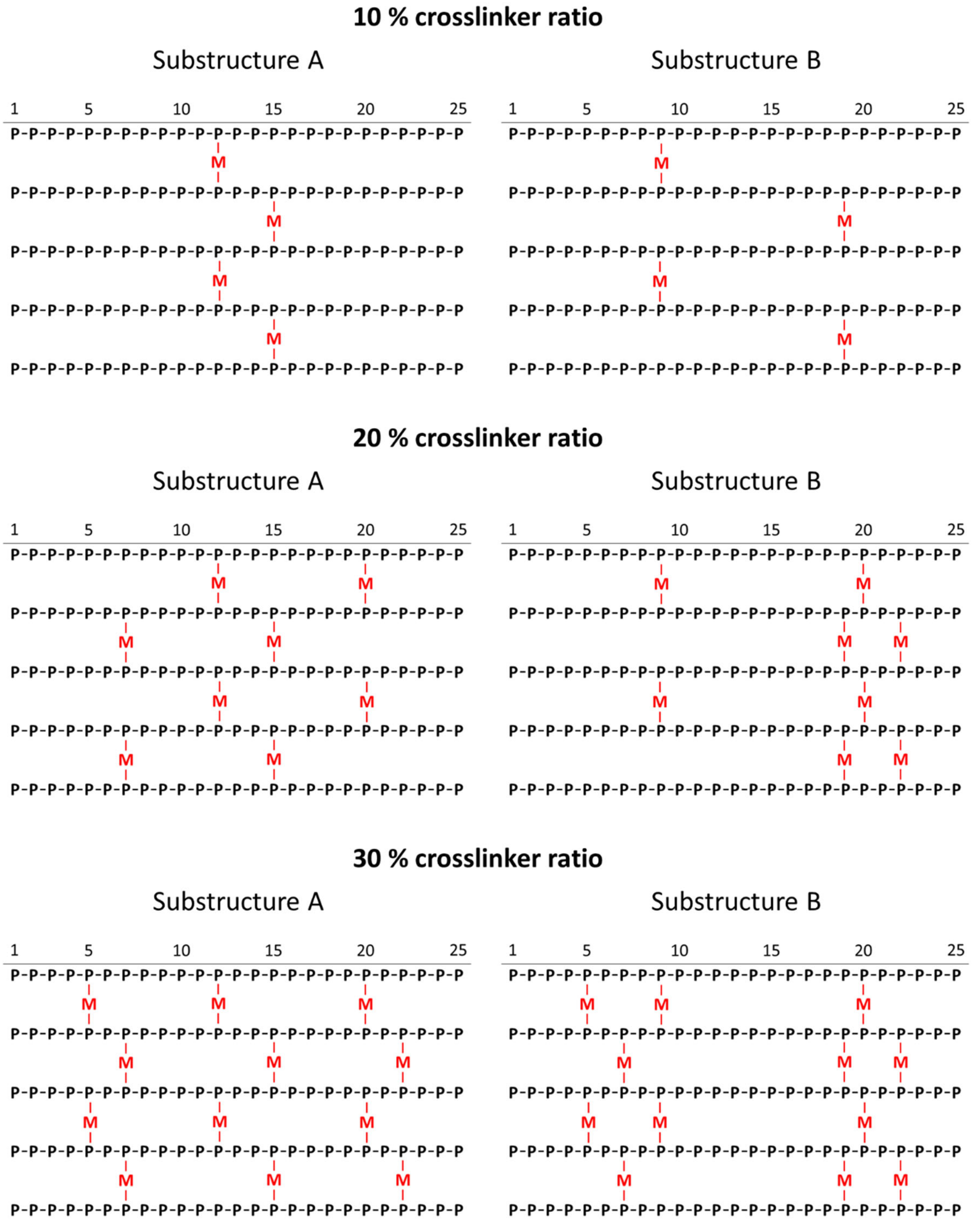

Figure S1. Scheme of quantity and distribution of the MA for each substructure of PVA-MA hydrogels.

Table S1. Assay to describe and optimize the capture and desorption of selected compounds by CLPHMA with ultrasonic agitation, at 20 ºC. Numbers in parentheses correspond to the coded values of the variables under study (ND: Not Detected).

Exp	Capture time [min]	Desorption time [min]	CLPHMA Composition [%]	pH of desorption solution [-]	mg/L of compound released per mg of CLPHMA				
					CFN	KCF	DMT	MTD	MTL
1	120 (1)	60 (-1)	30 (1)	3 (-1)	ND	0.0007	0.0010	ND	ND
2	10 (-1)	120 (1)	10 (-1)	9 (1)	0.0008	ND	0.0011	0.0025	ND
3	120 (1)	60 (-1)	30 (1)	3 (-1)	0.0008	ND	ND	0.0016	ND
4	120 (1)	60 (-1)	30 (1)	3 (-1)	0.0012	ND	ND	0.0048	ND
5	120 (1)	120 (1)	30 (1)	9 (1)	ND	0.0008	ND	0.0015	0.0003
6	10 (-1)	60 (-1)	10 (-1)	3 (-1)	ND	ND	ND	ND	0.0003
7	120 (1)	120 (1)	30 (1)	9 (1)	0.0008	ND	0.0014	ND	ND
8	10 (-1)	120 (1)	10 (-1)	9 (1)	0.0008	ND	ND	ND	ND
9	10 (-1)	120 (1)	10 (-1)	9 (1)	0.0028	ND	ND	0.0073	ND
10	10 (-1)	120 (1)	10 (-1)	9 (1)	ND	ND	ND	ND	0.0004
11	120 (1)	60 (-1)	30 (1)	3 (-1)	ND	0.0007	ND	ND	ND
12	120 (1)	120 (1)	30 (1)	9 (1)	0.0007	0.0008	ND	ND	ND
13	10 (-1)	60 (-1)	10 (-1)	3 (-1)	0.0005	ND	ND	ND	ND
14	10 (-1)	60 (-1)	10 (-1)	3 (-1)	0.0008	ND	ND	0.0014	ND
15	10 (-1)	60 (-1)	10 (-1)	3 (-1)	0.0011	ND	ND	0.0023	ND
16	120 (1)	120 (1)	30 (1)	9 (1)	0.0008	ND	ND	0.0043	0.0005
17	10 (-1)	90 (0)	10 (-1)	7.3 (0.43)	0.0005	ND	ND	0.0014	ND
18	65 (0)	90 (0)	20 (0)	7.3 (0.43)	ND	ND	ND	0.0015	ND
19	120 (1)	60 (-1)	30 (1)	7.3 (0.43)	0.0005	ND	ND	ND	ND
20	10 (-1)	60 (-1)	20 (0)	7.3 (0.43)	ND	ND	0.0013	0.0021	ND
21	65 (0)	120 (1)	30 (1)	7.3 (0.43)	0.0006	ND	ND	0.0019	ND
22	120 (1)	120 (1)	10 (-1)	7.3 (0.43)	0.0006	ND	ND	0.0013	0.0004

Table S2. Assay to describe and optimize the capture and desorption of selected compounds by CCCA microparticles with ultrasonic agitation at 20 ºC. Numbers in parentheses correspond to the coded values of the variables under study (ND: Not Detected).

Exp	Capture time [min]	Desorption time [min]	CCCA Composition [%]	pH of desorption solution [-]	mg/L of compound released per mg of CCCA				
					CFN	KCF	DMT	MTD	MTL
1	120 (1)	60 (-1)	56.5 (1)	3 (-1)	0.0022	0.0018	ND	0.0050	ND
2	10 (-1)	120 (1)	47.3 (-1)	9 (1)	0.0022	0.0032	0.0015	0.0048	ND
3	120 (1)	60 (-1)	56.5 (1)	3 (-1)	0.0025	0.0025	ND	0.0040	0.0035
4	120 (1)	60 (-1)	56.5 (1)	3 (-1)	0.0022	0.0023	0.0023	0.0041	0.0027
5	120 (1)	120 (1)	56.5 (1)	9 (1)	0.0020	0.0017	ND	0.0048	0.0017
6	10 (-1)	60 (-1)	47.3 (-1)	3 (-1)	0.0014	0.0017	0.0010	0.0035	0.0014
7	120 (1)	120 (1)	56.5 (1)	9 (1)	0.0015	0.0014	ND	0.0025	0.0016
8	10 (-1)	120 (1)	47.3 (-1)	9 (1)	0.0020	0.0014	0.0036	0.0015	0.0009
9	10 (-1)	120 (1)	47.3 (-1)	9 (1)	0.0014	0.0014	0.0010	0.0031	0.0003
10	10 (-1)	120 (1)	47.3 (-1)	9 (1)	0.0021	0.0024	ND	0.0024	0.0035

11	120 (1)	60 (-1)	56.5 (1)	3 (-1)	0.0022	0.0026	0.0020	0.0047	0.0016
12	120 (1)	120 (1)	56.5 (1)	9 (1)	0.0026	0.0028	0.0023	0.0035	0.0033
13	10 (-1)	60 (-1)	47.3 (-1)	3 (-1)	0.0021	0.0022	0.0010	0.0023	0.0034
14	10 (-1)	60 (-1)	47.3 (-1)	3 (-1)	0.0031	0.0015	ND	0.0070	0.0028
15	10 (-1)	60 (-1)	47.3 (-1)	3 (-1)	0.0018	0.0018	ND	0.0036	0.0011
16	120 (1)	120 (1)	56.5 (1)	9 (1)	0.0020	0.0018	ND	0.0018	ND
17	10 (-1)	90 (0)	47.3 (-1)	7.3 (0.43)	0.0014	0.0010	ND	0.0015	0.0009
18	65 (0)	90 (0)	52.4 (0.11)	7.3 (0.43)	0.0017	0.0013	0.0045	0.0043	0.0014
19	120 (1)	60 (-1)	56.5 (1)	7.3 (0.43)	0.0017	0.0017	0.0022	0.0038	0.0013
20	10 (-1)	60 (-1)	52.4 (0.11)	7.3 (0.43)	0.0028	0.0018	0.0032	0.0075	0.0009
21	65 (0)	120 (1)	56.5 (1)	7.3 (0.43)	0.0019	0.0017	0.0009	0.0029	0.0025
22	120 (1)	120 (1)	47.3 (-1)	7.3 (0.43)	0.0013	0.0011	0.0010	0.0049	0.0008

Table S3. Summary of the multifactorial multiple regression analysis for the analytes under study and their experimental variables, using CLPHMA, and ultrasonic agitation for extraction (Where A: capture time; B: desorption time; C: hydrogel composition; and D: pH of desorption solution) (NSS: Not Statistically Significant at the 95.0% or higher confidence level).

**Analyte**	**Influence**				**ANOVA model P-Value**
	**A**	**B**	**C**	**D**	
**CFN**	- (NSS)	+	-	-	0.7761
	Equation of the fitted model			R^2 (%)	
	CFN = 0.000611706 + 0.000196307*B - 0.00010894*C - 0.0000678702*D			9.43	
KCF	+	+	+	- (NSS)	0.2370
	Equation of the fitted model			R^2 (%)	
	KCF = 0.000135804 + 0.0000723359*A + 0.0000239794*B + 0.0000883222*C - 0.0000258484*D			26.52	
DMT	- (NSS)	-	+	+	0.6275
	Equation of the fitted model			R^2 (%)	
	DMT = 0.000204789 - 0.000243478*B + 0.000142168*C + 0.000337866*D			13.46	
MTD	-	+	+	- (NSS)	0.9349
	Equation of the fitted model			R^2 (%)	
	MTD = 0.00153501 - 0.000459895*A + 0.000502623*B + 0.000315214*C			4.49	
MTL	+	+	-	+ (NSS)	0.2689
	Equation of the fitted model			R^2 (%)	
	MTL = 0.0000821354 + 0.000109214*A + 0.0000403219*B - 0.000105851*C			25.09	

Table S4. Summary of the multifactorial multiple regression analysis for the analytes under study and their experimental variables, using CCCA, and ultrasonic agitation for extraction (Where A: capture time; B: desorption time; C: hydrogel composition; and D: pH of desorption solution) (NSS: Not Statistically Significant at the 95.0% or higher confidence level).

**Analyte**	**Influence**				**ANOVA model P-Value**
	**A**	**B**	**C**	**D**	
**CFN**	-	-	+	+ (NSS)	0.3405
	Equation of the fitted model			R^2 (%)	
	CFN = 0.00198997 - 0.000256563*A - 0.000147483*B + 0.000314512*C			22.25	
KCF	-	- (NSS)	+	-	0.8633
	Equation of the fitted model			R^2 (%)	
	KCF = 0.00185338 - 0.000141217*A + 0.000242778*C - 0.0000269197*D			6.92	
DMT	-	-	+ (NSS)	+	0.7030
	Equation of the fitted model			R^2 (%)	
	DMT = 0.0011691 - 0.000117156*A - 0.000907593*B + 0.00106634*D			11.42	
MTD	+	-	- (NSS)	+	0.4441
	Equation of the fitted model			R^2 (%)	
	MTD = 0.00378317 + 0.000222548*A - 0.00101861*B + 0.00042502*D			18.75	
MTL	- (NSS)	+	+	-	0.6669
	Equation of the fitted model			R^2 (%)	
	MTL = 0.00163456 + 0.000560102*B + 0.000518706*C - 0.000895016*D			12.39	
